# Allegheny County Veterinary Medical Association

**Published:** 1901-11

**Authors:** James A. Waugh

**Affiliations:** Secretary


### SOCIETY PROCEEDINGS.

### ALLEGHENY COUNTY VETERINARY MEDICAL ASSOCIATION.

A number of Pittsburgh and Allegheny veterinarians held an informal meeting at Hotel Schenly, on the evening of August 22, 1901, and after much deliberation perfected arrangements for the organization of a local association, which was completed at the same place on the evening of September 13, and the following officers duly elected: President, Dr. J. Stewart Lacock; Vice-President, Dr. N. Rectenwald; Treasurer, Dr. Chas. W. Boyd; Secretary, Dr. Jas. A. Waugh; Board of Directors, Dr. B. F. Bachman, Dr. D. C. Gearhart, Dr. J. C. McNeil, Dr. H. S. Richards, and Dr. J. E. Spindler; Charter Members, J. Stewart Lacock, H. S. Richards, J. E. Spindler, J. C. McNeil, N. Rectenwald, J. C. Kingan, H. N. Mayer, B. F. Bachman, Chas. W. Boyd, Jas. A. Waugh, H. Emery, David Martin, Geo. H. Dunn, G. P. Gilmore, F. Taylor. There was general discussion of existing conditions, and plans formulated for future work. Constitution and by-laws were adopted. The first regular monthly meeting was held October 2, 1901, at the office of Dr. J. E. Spindler. Dr. A. W. Hinman, of Braddock, and Dr. John Spohn, of Homestead, were elected regular members; and Dr. F. W. Ainsworth and Dr. Benj. Howes, of the Bureau of Animal Industry, were elected honorary members. These meetings have been favored with a large attendance, much sociability, and great interest in the welfare of the profession.

JAMES A. WAUGH, V.S.,
Secretary.

